# Switching to letrozole or exemestane improves hot flushes, mood and quality of life in tamoxifen intolerant women

**DOI:** 10.1038/sj.bjc.6604323

**Published:** 2008-04-08

**Authors:** R Thomas, M Williams, C Marshall, L Walker

**Affiliations:** 1 Primrose Oncology Research Unit, Bedford Hospital NHS Trust, Kempston Road, Bedford MK4 9DJ, UK; 2 Cranfield Health, Cranfield University, Cranfield, Bedfordshire MK43 0AL, UK; 3 Addenbrooke's Hospital (Cambridge University) NHS Trust, Hill's Road, Cambridge CB2 2QQ, UK; 4 Institute of Rehabilitation, The University of Hull, Anlaby Road, Hull HU3 2PG, UK

**Keywords:** aromatase inhibitors, breast cancer, quality of life, tamoxifen intolerance

## Abstract

We report an open-label, prospective, crossover study involving 184 post-menopausal women experiencing hot flushes on adjuvant tamoxifen (T). Six weeks after switching to an AI, the primary end point, hot flush score, improved by 47.3% (*P*<0.001) compared to those reported on T. The mean mood rating scale (MRS) score improved by 9.7% (*P*=0.01). The total mean combined FACT (b+es) score improved from 134.2 (95% CI ±2.96) to 143.5 (95% CI ±2.96 <0.001), and the endocrine subscale improved by 9.8% from 51.73 (95% CI ±1.38) to 57.34 (CI ±1.38, *P*<0.001). At 6 weeks, significantly more women chose to remain on an AI: 133 (72%), *vs* 40 (22%) (*P*<0.001) preferring T. At 3 months, 107 (58%) preferred to remain on an AI, 55(30%) on T, and 22 (12%) withdrew. The overall arthralgia rate at 3 months was 47% on AI and 30% on T (*P*=0.001). In all 182 (99%) women reported appreciating the opportunity to experience both drugs. These data suggest that if patients suffering significant adverse effects on T are given the opportunity to try an AI, this empowers them to prioritise relative side-effects, improving wellbeing in a significant proportion. These data also highlight the need for hospital follow-up in this intolerant cohort.

The routine adjuvant endocrine standard of care for post menopausal women with oestrogen receptor positive breast cancer has shifted towards including an aromatase inhibitors (AI), following the evidence presented in five international trials demonstrating improved disease-free survival with AI containing regimens, compared to tamoxifen alone (Baum, 2001; Mouridsen *et al*, 2001; Coombes *et al*, 2004; Boccardo *et al*, 2005; Jakesz *et al*, 2005).

It is, however, unlikely that all post menopausal women, particularly those with a good prognosis, where the relapse rate is low on either drug, have a clinically relevant advantage (Baum, 2001). Subgroups of patients have been identified who would benefit the most from AIs over tamoxifen, and particularly benefit from initial selection rather than sequencing a switch at 2 years. These include pathological prognostic factors which predict a higher and earlier relapse rate, such as histological grade, presence of vascular invasion, tumour size and number of positive axillary nodes (Mauriac *et al*, 2007); Biological molecular markers such a Cerb2, Ki67, CYP-2D6, level of oestrogen receptor positivity, progesterone receptor negativity (Ellis *et al*, 2001; Mauriac *et al*, 2007); Patient-related factors such as a history of thromboembolic disease, risk of uterine carcinoma or osteoporosis (Ellis *et al*, 2001; Lonning *et al*, 2005; Mauriac *et al*, 2007). The roles played by quality of life, tolerance and patient preference in the choice for either AI or tamoxifen; however, has not been formally established previously.

Menopausal symptoms and overall quality of life have been extensively investigated within adjuvant breast cancer studies (Baum, 2001; Coombes *et al*, 2004; BIG 1-98 Collaborative Group, 2005; Boccardo *et al*, 2005; Jakesz *et al*, 2005), with particular scrutiny within the ATAC (Fallowfield *et al*, 2004a) and IES (Fallowfield *et al*, 2006) trials. In these studies arthralgia and vaginal dryness was more prevalent with AIs and night sweats and vaginal discharge with tamoxifen but there was no difference in overall quality of life. Most women in these studies, fortunately, had no significant menopausal symptoms on either treatment. In daily practise, however, the clinically more relevant but unanswered question, was whether switching to an AI in the 30% already intolerant of tamoxifen improved or compromised quality of life.

This study assessed the short-term effect on hot flushes, mood and quality of life, and the longer-term effect on arthralgia and preference for tamoxifen or AI, subsequent to giving patients personal experience of both drugs within a prospective crossover design. The results aimed to supplement existing histological and patient oriented factors, to aid the decision for either early switching to AI or continuing tamoxifen.

## METHODOLOGY

This open-label, crossover trial assessed post menopausal women with oestrogen receptor positive breast cancer, experiencing troublesome hot flushes while taking adjuvant tamoxifen. One hundred and eighty four women were recruited between March 2005 and February 2007 at the Primrose Oncology Research Unit, Bedford Hospital, UK and Russells Hall Hospital, Dudley, UK. Ethics approval was sought, and given, for this study.

### Patient selection

All 184 women gave written informed consent and had been taking adjuvant tamoxifen for at least 3 months prior to trial entry. The average age was 58.8 years (range, 40.6–88.7 years). The average time on tamoxifen was 15.5 months (3–62 months). The average time from diagnosis to switching to an AI was 20.34 months (range, 9–88 months), 54 (29%) had received adjuvant chemotherapy. Patients had a histological or cytological evidence of breast cancer which demonstrated oestrogen-receptor (ER) positivity and were post menopausal at the time of initiation of AI, as defined by; no spontaneous menses for at least 2 years, or spontaneous menses within the past 2 years, but amenorrhoeic for at least 12 months and oestrodiol, LH and FSH values according to the definition of postmenopausal normal range of the laboratory involved; or bilateral oophorectomy or radiation castration and amenorrheic for at least 3 months or medical oophorectomy with administration of LHRH agonists. In all 48 (26%) were deemed to be menopausal when they first started taking adjuvant tamoxifen patients but then met the definition of postmenopausal at trial entry. Patients scored >3 on the NCI toxicity score or had average of 14 significant hot flushes per week. All women had tried, or were offered, lifestyle advice for hot flushes, including our written guidance sheet (‘coping with hot flushes’ – available on www.cancernet.co.uk). No women were taking venlafaxine or clonidine at trial entry. Many women had tried over the counter remedies for hot flushes (evening Primrose Oil, Black Cohosh) but were excluded if these started within 1 month before the trial period, otherwise these were allowed as long as they continued unchanged.

### Tolerance assessment

#### Hot flushes score

The hot flushes diary (HFD) and scoring system used as the primary end point in this study were originally developed and validated by Sloan *et al* (2001). The HFD is an internationally accepted tool for evaluating and comparing hot flushes strategies, and has been used in a number of randomised trials such as those involving venlafaxine, clonidine and fluoxetine (Loprinzi *et al*, 2000, 2002; Loibl *et al*, 2007). The scoring system gives an indication of frequency and severity by grading hot flushes into mild, moderate, severe and very severe, over a 24-h period. In this study, as these forms were self completed by patients at home, they were also given a leaflet defining the grades of hot flushes to help them complete the HFD. As recommended in previous validation studies, despite these grading systems, it was still ultimately the patient's own interpretation of severity that was scored. In other words, if a woman told us she had a severe hot flush, we did not feel that it was appropriate for us to tell her it was only a mild hot flush.

#### Quality of life

The Functional Assessment of Cancer Therapy (FACT), breast version with Endocrine Symptom add-ons (FACT-B+ES) was used (Cella, 1997). This questionnaire covers physical as well as psychological symptoms, and includes a component designed specifically for women with breast cancer who receive hormonal therapy. The FACT-ES has been validated in the advanced breast cancer setting, and has been shown to have good internal consistency, reliability, patient acceptability and sensitivity to clinically significant change (Cella, 1997). It has been used in the majority of large adjuvant AI studies (Coombes *et al*, 2004; Fallowfield *et al*, 2004a).

#### The Mood Rating Scale

The Mood Rating Scale (MRS) is a self-reported measure of normal mood, which consists of six 150 mm visual analogue subscales with defined anchor points. The subscales are: tense-relaxed, sad-happy, tired-energetic, confused-clear headed, irritable-easy-going, unsure-confident (Anderson *et al*, 2000). The six subscales can be summated to obtain a single total score. Reliability and validity have been found to be good (Anderson *et al*, 2000), and the scale has been shown to be responsive to the effects of cancer therapies (Walker *et al*, 1997, 1999a), psychological interventions (Walker *et al*, 1999b) and work-induced stress. It takes approximately one minute to complete (Anderson *et al*, 2000).

#### The patient preference questionnaire

The patient preference questionnaire was originally developed by one of the trial committee as a tool to formalise and grade the strength of a patient's choice (Thomas *et al*, 1999). It has been used as an end point in a subsequent crossover trial involving AIs, and consistently correlated with quality of life and degree of side effects (Thomas *et al*, 2004). Patients are asked to indicate which drug they tolerated better overall, the reasons for this choice, the level of confidence in their decision, and their general views on the appropriateness of being asked the question. Patient preference has been used in a number of other trials on subjects ranging from oral *vs* i.v. chemotherapy, to choices between chemotherapy and hormone therapies (Liu *et al*, 1997; Young *et al*, 1999; Fallowfield *et al*, 2004b).

#### Arthralgia grading system

The NCI system was used, which grades severity into mild, moderate, severe and very severe (National Cancer Institute, 1999).

### Study design

Following written informed consent obtained while taking tamoxifen 20 mg per day, women completed a 1-week HFD, the FACT-B+ES questionnaire, an MRS and an NCI arthralgia grading scale. They were then switched to an AI. The same questionnaires plus a patient preference questionnaire (PPQ) were given to patients for completion at home, and they were asked to post back to the trial centre (to minimise social compliance effects). For the first 104 patients in the study, letrozole 2.5 mg per day was prescribed: the next 80 patients were given exemestane 25 mg per day. The women either continued on their prescribed AI or changed back to tamoxifen, based on their patient preference and quality of life questionnaires. A further arthralgia NCI grading score and PPQ was completed by patients at 3 months. At any stage after the initial 6-week trial, women were given the opportunity to switch back to tamoxifen if they subsequently developed troublesome side effects on their AI, and in each case the trial office was informed. All women who chose an AI were given ‘Lifestyle & AI’ guidance sheet (available from www.cancernet.co.uk/bonehealth.htm) and their names were entered into the standard bone density surveillance programme.

#### Statistical considerations

The methodology and analysis was conducted at Cranfield University. The primary end point was the hot flush score (HFS intensity × number as assessed by the HFD). It was intended that both the letrozole and exemestane phases were statistically and independently powered.

Sample size consideration

The number of patients for the first (letrozole) phase was determined using classic power analysis characteristics, based on previous studies in populations of patients with hot flushes (Loprinzi *et al*, 2000, 2002; Sloan *et al*, 2001). In these trials, with 50 patients per group, the studies had 80% power to detect differences in average hot flush activity of 0.6 s.d. using a standard two-sample *t*-test with a two-sided type I error rate of 5%. Hence, 50 patients per treatment arm provided an 80% power to detect an average shift of 1.2 hot flushes per day or a hot flush score of three units per day. Smaller differences were regarded as not clinically significant. Other trials using similar methodologies had statistically proven benefits of hot flush remedies using similar patient numbers (Loprinzi *et al*, 2000, 2002; Sloan *et al*, 2001). The use of a crossover design, in this study, increased power as patients acted as their own controls.

Placebo effect consideration

It has been recognised in previous hot flush studies that a placebo effect may reduce hot flush activity up to approximately 25%, that is, to 75% of baseline (Loprinzi *et al*, 2000, 2002; Sloan *et al*, 2001). In view of this placebo effect, a further 30 patients were added, or required, to ensure a minimum of 80% power to detect a difference in average hot flush activity of 0.6 s.d. (a reduction of 25% plus 1.2 hot flushes per day or a reduction of the score by 25% plus a score of three units) with a type 1 error rate of 5%. That is, if a woman has six hot flushes per day, a reduction to an average of 3.3 would be regarded as significant. As an extra reassurance in the first letrozole phase, a further 20 patients were added.

Because an interim analysis of this first phase (letrozole) comfortably reached statistical significance, the second (exemestane) phase adhered to the precise power recommendation of 80 patients.

Statistical analysis

The HFS, MRS and FACT-B+ES were analysed using parametric analysis of variance. This was considered the most robust method, as even with non-normal data, the estimated least square means and their confidence intervals of these data were sound. Retrospective analysis of errors showed that there was no serious departure from normally distributed errors, and there was constant variance across all the predicted means. The total FACT (b+es), its six subscales were also analysed using the analysis of variance method with all the considerations made according to the original FACT guidance manual. Similarly, the MRS data was analysed using analysis of variance. Pearson *χ*^2^ tests were used to analyse the PPQ and arthralgia scores.

## RESULTS

One hundred and eighty-four patients entered the study. One patient had demonstrated signs of relapse by 6 weeks, and by 3 months, five in total had relapsed. All completed their questionnaires and were included in the analysis. The data was analysed in three separate groups; all patients combined; the letrozole phase alone, and the exemestane phase alone (Tables 1, 2 and 3). During the recruitment period for this trial, only two further eligible patients declined trial entry, suggesting the trial cohort closely reflected routine oncology practise.

### Hot flushes

**Figure 1:**
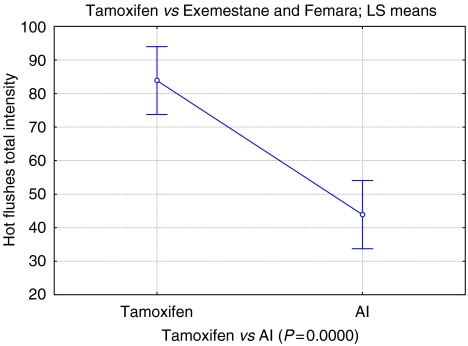
Tamoxifen *vs* AI hot flushes total intensity. Vertical bars denote 0.95 confidence intervals.

The severity of hot flushes, following 6 weeks AI, almost halved from those experienced on tamoxifen, as measured by the HFS (47.3%, *P*<0.001) (Table 1, Figure 1). Among the 133 (72%) who chose AI, the difference in HFS was understandably even larger at 6 weeks (*P*<0.0001).

### Quality of life

**Figure 2:**
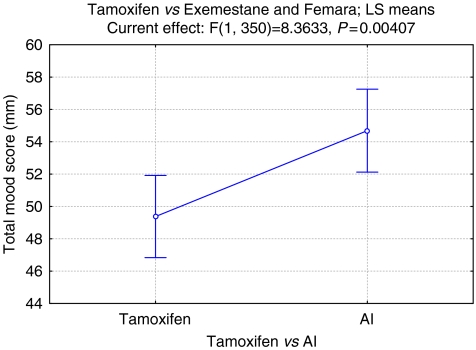
Tamoxifen *vs* AI total mood score (mm). Vertical bars denote 0.95 confidence intervals.

**Figure 3:**
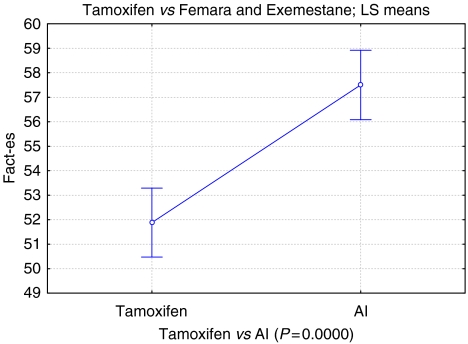
Tamoxifen *vs* AI fact-es. Vertical bars denote 0.95 confidence intervals.

The total mean combined FACT (b+es) score improved by 6.5%, from 134.2 (95% CI ±2.96), to 143.5 (95% CI ±2.96, *P*<0.001). The endocrine subscale improved by 9.8% from 51.73 (95% CI ±1.38) to 57.34 (95% CI ±1.38, *P*<0.001). All other subscales also reached significance (Table 1, Figures 2 and 3).

### Mood

**Figure 4:**
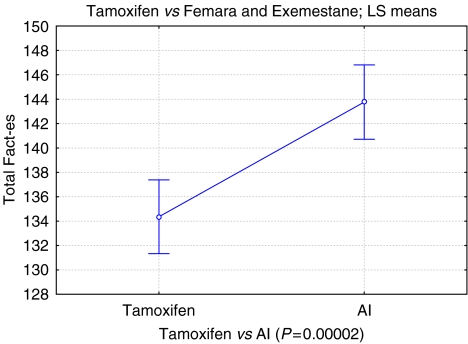
Tamoxifen *vs* AI total fact-es. Vertical bars denote 0.95 confidence intervals.

Total MRS scores, improved by 9.7% from 49.4 (95% CI ±2.48) to 54.7 mm (95% CI ±2.48, *P*=0.005) (Table 1, Figure 4).

### Patient preference

At six weeks, 133 (72%) preferred to remain on an AI as they felt their menopausal symptoms and other side effects were better. Forty (22%) patients felt that they were better on tamoxifen and 11 (6%) discontinued adjuvant hormones altogether as they decided the side effects on either hormone did not justify their adjuvant benefits. At or after 3 months, 107 (58%) remained on an AI, 55 (30%) stayed on tamoxifen, and 19 (10.3%) of those originally choosing an AI resumed tamoxifen, primarily due to the development of troublesome arthralgia. One switched from tamoxifen to exemestane. Twenty-two (12%) women, following discussion chose to discontinue hormone therapy altogether (Table 2).

### Arthralgia

The overall arthralgia rate among the 173 still in the study after 6 weeks (11 had withdrawn), and choosing an AI was 47% during AI administration, and those on tamoxifen was 30% (Pearson *χ*^2^, *P*=0.001). This was mainly due to the difference in severe arthralgia (T 0% *vs* AI 13%), as there was no significant difference in the mild to moderate rates (Table 3). The same trends were seen in the individual analysis of letrozole and exemestane.

One hundred and eighty (98%) patients indicated on the final questionnaire that they appreciated the opportunity to experience both drugs, and to be able to make choices for themselves between hot flushes and arthralgia.

Although not initially part of the analysis plan, a direct comparison of letrozole *vs* exemestane showed no difference between them in any predetermined trial end point category.

## DISCUSSION

Menopausal symptoms, particularly hot flushes, significantly impair quality of life in women with breast cancer (Carpenter *et al*, 1998; Loprinzi *et al*, 2002; Fallowfield *et al*, 2004b; Loibl *et al*, 2007), who have a significantly higher incidence of these symptoms than the general population (Carpenter *et al*, 1998). Premenopausal women may have undergone premature menopause from chemotherapy or ovarian ablation, and post menopausal women can endure sudden interruption of their hormone replacement therapy (HRT), causing a recognised rebound to severe climacteric symptoms (Jensen and Christiansen, 1983). On top of this, they are prescribed drugs such as tamoxifen and aromatase inhibitors, which make them worse. The most widely used non-oestrogenic therapeutic interventions for hot flushes in women with breast cancer, include progestogens, clonidine and venlafaxine (Carpenter *et al*, 1998; Loprinzi *et al*, 2000, 2002; Sloan *et al*, 2001; Loibl *et al*, 2007). A low dose of progesterone such as medroxyprogesterone acetate; however, can cause weight gain and increase the risk of thromboembolus, and the longer term risks of its addition to tamoxifen are uncertain (Carpenter *et al*, 1998). Clonidine has limited success but can cause troublesome adverse effects of its own (Goldberg *et al*, 1994). Venlafaxine has recently been shown to be more affective than clonidine, but women are apprehensive about starting long term antidepressants (Loprinzi *et al*, 2000; Loibl *et al*, 2007). Lifestyle advice such as sensible clothing and avoiding stimulants can be helpful (Sloan *et al*, 2001), and hence all patients in this study received our written lifestyle guidance (Cancernet-UK, 2008). Dietary supplements such as evening primrose oil are popular, but lack evidence of benefit. Likewise, complementary interventions such as acupuncture, reflexology and massage, remain unproven remedies (Richardson *et al*, 2005). Randomised controlled trials to evaluate herbal therapies and phytoestrogen supplements have shown limited benefit, and the consensus among oncologists is that, if they do work, their oestrogenic effect may be detrimental in terms of risk of tumour relapse (MacGregor *et al*, 2005).

The quality of life benefits of AIs over tamoxifen were not demonstrated within the five large international, published adjuvant breast cancer studies (Baum, 2001; Coombes *et al*, 2004; BIG 1-98 Collaborative Group, 2005; Boccardo *et al*, 2005; Jakesz *et al*, 2005), even though the ATAC (Fallowfield *et al*, 2004a) and IES trials (Fallowfield *et al*, 2006) had further specific scrutiny. There are two possible explanations for this. Firstly in the above studies, a significant proportion of women fortunately tolerated both tamoxifen and AI well, so any difference in quality of life between the two groups was diluted by the ‘no side effect’ cohort; Second, side effects are more prevalent with AIs (arthralgia and vaginal dryness) in terms of a quality of life analysis, directly balance those more likely in the tamoxifen group (night sweats and vaginal discharge). The fundamental difference in the cohort of the women evaluated in this paper, is that they all had significant hot flushes at trial entry. The subsequent 53.7% improvement in hot flushes, in terms of quality of life and preference, in these patients outweighed the 17% deterioration in arthropathy.

Another possibility of the significant findings of this study maybe, albeit unlikely, as a weakness in its open design. Blinding would have avoided the influence of external sources such as media reports on the benefits of AIs, but the cost of repackaging was prohibitive. Although the benefits of switching were over and above the anticipated placebo effect, a causal effect should ideally be confirmed by a double-blind, randomised trial. However, it was in the patient's interests to be honest about side effects because they would be taking the medication after the 6-week trial period. Nevertheless, media activity could have biased a small proportion of patients towards AI especially in the group who did not notice much difference between the two drugs. However, the finding that 30% of women chose tamoxifen at 6 weeks or later, and 12% chose to stop altogether, suggest that patients were more than capable of weighing up the complex issues of risks, benefits and quality of life, provided they were empowered with the appropriate experience. Furthermore, citations in the UK media, referring to AIs (Table 4), increased over the duration of the study (208 letrozole phase, 303 exemestane) (Cision Public Relations, 2005–2007) whereas the percentage of patients choosing an AI in the two phases was the same, suggesting media reports were not a strong influence on their decision-making.

Another criticism of the open design may potentially be inadvertent physician bias. To diminish this risk, all questionnaires were completed outside the clinic environment, and returned to the hospital by post. There did, however, appear to be a change in physician's attitudes to the trial entry over the 2-year recruitment period. Despite the eligibility criteria being the same, the intensity of hot flushes (HFS) at trial entry was slightly lower in the later, exemestane group at entry. This probably reflected changing attitudes in clinicians and patients to switching to AIs as the international evidence emerged. This trend, although not affecting the statistical power, results and conclusion did highlight that the quality of life benefits of switching applies both to patients who had either severe hot flushes (HFS mean 97) or moderate hot flushes (HFS mean 70.7).

Despite these caveats in trial design, its strength is that the issues raised very much reflect those confronting patients with breast cancer and oncologists on a daily basis. The reality that only two tamoxifen-intolerant women declined trial entry strongly suggests that these findings reflect routine clinical practise. It was also reassuring to report that 99% of patients in the study indicated that they greatly appreciated the opportunity to experience both drugs: implementing a crossover manoeuvre into clinical practise is likely to be widely accepted and appreciated. This trial also highlights patient's capacity and enthusiasm, if empowered with personal experiences, to choose which drug best fits their own daily lives, particularly trading off lower hot flushes for higher arthralgia. The ability to share decisions with their clinicians has previously been a factor associated with improved psychological wellbeing, satisfaction and compliance (Thomas *et al*, 1999, 2000, 2004).

Although there was a statistical benefit in favour of AIs, it must be emphasised that a significant minority of the women felt worse on AIs. This resulted in 10% switching back to tamoxifen and 12% of participants withdrawing from hormones altogether, and, no doubt, another group who had considered switching back, but decided to remain on their chosen drug. In either scenario, patients required considerable discussion with their clinicians within longer and more frequent consultations, which prevented referral for follow up back to their general practitioners, and has resource implications for breast clinics.

To conclude, despite the limitations of an open design, these data suggest that patients suffering significant side effects on adjuvant tamoxifen should be given the opportunity to try an AI, as in this study the majority had an improved level of hot flushes, quality of life and mood. This trial highlights patient's capacity and enthusiasm, if empowered with personal experiences, to share decisions with their clinicians and choose which drug best fits their lives, particularly trading off lower hot flushes for higher arthralgia. This manoeuvre is likely to lead to a better quality of life for women receiving adjuvant hormone therapies, but will require greater resources for more frequent and more in-depth follow-up consultations.

## Figures and Tables

**Table 1:** Results of hot flushes, mood and qol before and after 6 weeks of AI

**(*n*=104)**	**Tamoxifen**	**Letrozole**	**% Difference (*P*)**
HFS	97.0±13.13^a^	52.1±13.13	46.3%^b^ (*P*=0.001)
Total FACT-b+es	132.7±3.92	142.1±3.92	6.7% (*P*=0.01)
FACT-es	50.65±1.83	56.34±1.83	10.1% (*P*=0.01)
MRS (mm)	49.6±3.29	54.9±3.29	9.6% (*P*=0.004)
			
**(*n*=80)**	**Tamoxifen**	**Exemestane**	**Difference (*P*)**
HFS	70.7±8.22	36±8.22	49% (*P*<0.001)
Total FACT-b+es	136±4.49	145.4±4.49	4.9% (*P*=0.001)
FACT-es	53.12±2.09	58.7±2.09	9.5% (*P*<0.001)
MRS (mm)	49.1±3.78	54.5±3.78	9.6% (*P*=0.005)
			
**(*n*=184)**	**Tamoxifen**	**Combined AI**	**Difference (*P*)**
HFS	85.6±9.90	45.1±9.90	47.3% (*P*<0.001)
Total FACT-b+es	134.2±2.96	143.5±2.96	6.5% (*P*=0.001)
FACT-es	51.73±1.38	57.34±1.38	9.8% (*P*<0.001)
MRS (mm)	49.4±2.48	54.7±2.48	9.7% (*P*=0.01)

a Figures represent 95% confidence intervals (± 2 s.e. from the mean).

b % difference is subtraction of mean on T from the mean on AI/by the highest of the two values (e.g. 97–52.1/97=46.3%).

**Table 2:** Patient's preference for adjuvant hormone therapy at 6 weeks and ⩾3 months

**Start (*n*=104)**	**Tamoxifen (T)**	**Letrozole (L)**	**Difference^a^ (*P*)**
At 6 weeks 8 (8%) withdrew^b^	26% (25/96)	74% (71/96)	48% (*P*<0.001)
⩾3 months 16 (15%) withdrew^b^	31% (27/89)	69% (61/89)	38% (*P*<0.001)
			
**Start (*n*=80)**	**Tamoxifen (T)**	**Exemestane (E)**	**Difference (*P*)**
At 6 weeks 3 (4%) withdrew^b^	19% (15/77)	81% (62/77)	62% (*P*<0.001)
⩾3 months 6 (7%) withdrew^b^	38% (28/74)	62% (46/74)	24% (*P*=0.036)
			
**Start (*n*=184)**	**Tamoxifen (T)**	**Combined AI**	**Difference (*P*)**
At 6 weeks 11 (6%) withdrew^b^	23% (40/173)	77% (133/173)	54% (*P*<0.001)
⩾3 months 22 (12%) withdrew^b^	34% (55/162)	66% (107/162)	32% (*P*<0.001)

a The % difference is subtraction of percentage on T from the percentage on AI.

b 22 (12%) patients withdrew by 3 months, 5 (2.7%) because they had relapsed, 17 (9.2%) withdrew for intolerance. A further 19 (10.3%) switched back to tamoxifen from an AI (4 from L, 15 from E), two patients later switched to AI at 3 months (1 L, 1 E).

**Table 3:** Arthralgia rate at or after 3 months of the chosen hormone therapy

**Arthralgia-3 months**	**Chosen hormone**		
***n*=173 after 6 weeks^a^**	**Tamoxifen (*n*=40)**	**Both AI (*n*=133)**	**Difference (*P*)**
None	28 (70%)^b^	70 (53%)	17% (*P*=0.001)
Mild arthralgia	10 (25%)	30 (22%)	3% (ns)
Moderate arthralgia	2 (5%)	16 (12%)	7% (ns)
Severe arthralgia	0 (0%)	17 (13%)	13% (*P*=0.05)
Overall arthralgia	12 (30%)	63 (47%)	17% (*P*=0.001)

a Eleven of the 184 patients had withdrawn at 6 weeks.

b For descriptive purposes the percentages of arthralgia for T or AI written in each colomn is based on n for each column whereas for the Pearson *χ*^2^ analysis *n* was 173.

**Table 4:** UK media citations for aromatase inhibitors for the duration of the study

**Key search words**	**01/03/05–30/11/05**	**01/12/05–28/02/07**
Arimidex	127	129
Aromasin	40	67
Femara	41	107
Any AI	208	303

Courtesy of Cision Public Relations Ltd.
